# Reproductive Factors and Serum Uric Acid Levels in Females from the General Population: The KORA F4 Study

**DOI:** 10.1371/journal.pone.0032668

**Published:** 2012-03-13

**Authors:** Doris Stöckl, Angela Döring, Barbara Thorand, Margit Heier, Petra Belcredi, Christa Meisinger

**Affiliations:** 1 Institute of Epidemiology II, Helmholtz Zentrum München, German Research Center for Environmental Health, Neuherberg, Germany; 2 Department of Obstetrics and Gynaecology, Campus Grosshadern, Ludwig-Maximilians-University, Munich, Germany; 3 Institute of Epidemiology I, Helmholtz Zentrum München, German Research Center for Environmental Health, Neuherberg, Germany; 4 Central Hospital of Augsburg, MONICA/KORA Myocardial Infarction Registry, Augsburg, Germany; FuWai Hospital, Chinese Academy of Medical Sciences, China

## Abstract

**Objective:**

Hyperuricemia is associated with an increased risk of metabolic and cardiovascular diseases. There are pronounced sex differences in the levels of uric acid. It is largely unknown whether or not reproductive parameters which induce hormonal changes are responsible for this. We examined if there are associations between reproductive parameters and uric acid levels in a female population-based sample.

**Methods:**

In this cross-sectional analysis, data of 1530 women aged 32 to 81 years participating in the KORA F4 study, conducted between 2006 and 2008 in Southern Germany were used. Reproductive parameters were obtained by standardized interviews. Uric acid levels were tested by the uricase method. The whole study sample and stratified in pre- and postmenopausal women was analyzed.

**Results:**

Menopausal status and earlier age at menarche were associated with higher serum uric acid levels (age-adjusted: p-values 0.003, <0.001 respectively; after multivariable adjustment, including BMI: p-values 0.002, 0.036). A history of oral contraceptive use showed an association with uric acid levels only after multivariable adjustment (p-value 0.009). Hot flushes showed an association with uric acid levels only after age-adjustment (p-value 0.038), but lost significance after adding other confounders. Other reproductive factors, including parity, current or ever use of hormone replacement therapy, current use of oral contraceptives, hysterectomy, bilateral oophorectomy, or depressive mood related to menopausal transition were not associated with uric acid levels.

**Conclusions:**

Postmenopausal status, earlier age at menarche and a history of oral contraceptive use were independently associated with higher serum uric acid concentrations in women from the general population. Further studies, especially longitudinal population-based studies investigating the relationship of female reproductive parameters with uric acid levels are necessary to confirm our findings.

## Introduction

Hyperuricemia is not only the main risk factor for gout in women [1], [2], but also associated with chronic diseases, such as the metabolic syndrome [3], [4], type 2 diabetes [5] and cardiovascular diseases [6], [7] in women as well as in men with some sex-related dissimilarities [8]. There are pronounced sex-specific aspects in the levels of uric acid [2], [9]. In women compared to men uric acid levels are lower in younger ages, but the difference becomes smaller with older ages, and especially after menopause this gender difference is lost [10], [11]. The main factor for this is presumed to be a female hormone influence. But the underlying pathways and their importance have been sparsely examined. During pregnancy, uric acid levels initially decrease compared to pre-pregnancy values and in the second half of the pregnancy they increase and are higher than before pregnancy [12], pointing towards hormonal influences on uric acid levels. Genetic factors are known to influence uric acid levels, with a stronger effect in women than in men [9]. Some reproductive events which occur over the life span of women are influenced by genetic traits as well, like age at menarche [13].

Some studies have examined selected reproductive factors and its influence on gout prevalence. Earlier menopause [14], [15] was shown to be a risk factor for gout while hormone replacement therapy seems to modestly reduce the risk of gout [15]. Other research has examined the menopausal status or menopausal transition and its effect on uric acid levels [10], [11], [16]. It could be shown that serum uric acid levels rise after menopause. Whether this is simply an age effect, or if the hormonal changes during the menopause have a direct effect on uric acid levels needs further exploration. Postmenopausal hormone replacement therapy reduces uric acid levels [10], [17], [18]. Thus, prior studies have investigated the effect of selected reproductive parameters on uric acid levels to some extend, with a certain lack of population-based studies. In addition, to the best of our knowledge, the role of some reproductive factors such as age at menarche have not been previously studied.

Thus, the aim of this study was to investigate whether there is an association between a variety of reproductive parameters and uric acid levels in women from the general population.

## Methods

### Subjects

The KORA F4 study (2006–2008) contains 7-year follow-up data of the KORA S4 study (1999–2001), a population-based health survey conducted in the city of Augsburg and the two surrounding counties. For the S4 baseline study, a total sample of 6640 subjects was drawn from the target population consisting of all German residents of the region aged 25 to 74 years. The study design, sampling method and data collection have been described in detail elsewhere [8], [19].

Of the 4261 participants in S4, 3080 took part in the F4 follow-up study. Participants were not included in F4 if they had died in the time in-between (n = 176, 4%), relocated outside the study region, were lost to follow-up (n = 206, 5%) or had demanded deletion of their contact data (n = 12, 0.2%). Of the remaining 3867 eligible persons, 174 could not be contacted, 218 were unable to participate in the F4 study because they were too ill or had no time, and 395 did not want to take part in the follow-up. The resulting response was 79.6%. The current study was restricted to 1594 female subjects, aged 32 to 81 years at follow-up.

We excluded all women for whom no or incomplete information on uric acid levels (n = 8) or any of the covariables was available (n = 56). The final analysis thus included 1530 women.

### Ethics Statement

The investigations were carried out in accordance with the Declaration of Helsinki, including written informed consent of all study participants. The study was approved by the Ethics Committee of the Bavarian Medical Association.

### Data collection

Information on socio-demographic variables, physical activity level, medication use, alcohol consumption, smoking habits and reproductive history was obtained by trained medical staff during a standardized face-to-face interview. All study participants were asked to bring original packaging of their medications used during the last 7 days before the study examination. Pharmaceutical control numbers, names etc. were recorded and ATC (Anatomical Therapeutic Chemical Classification System) codes assigned accordingly, including uricosuric and uricostatic medication. In addition, all study participants underwent a standardized medical examination. All measurement procedures have been described in detail elsewhere [8]. Anthropometric measurements were taken after the participants had removed their shoes, heavy clothing and belts. Body height was measured to the nearest 0.1 cm and weight to the nearest 0.1 kg. BMI was calculated as weight [kg] divided by height^2^ [m^2^]. Waist circumference (WC) was measured at the level midway between the lower rib margin and the iliac crest. Actual hypertension was defined as blood pressure values greater than 140/90 mmHg or the use of antihypertensive medication, given that the participants were aware of being hypertensive. Participants were classified as active during leisure time if they regularly participated in sports in the summer and winter and if they were active for >1 h/wk in either season. Women who consumed on average more than 20 g alcohol per day were regarded as heavy alcohol drinkers.

### Clinical chemical measurements

A venous blood sample was obtained from all study participants while sitting. Serum levels of uric acid were analysed by the uricase method (enzymatic color-test, URCA Flex), total cholesterol and high-density lipoprotein cholesterol (HDL-C) with a CHOD-PAP method (CHOL Flex) and serum creatinine with the analyzer Dimension RxL, all assays from Dade Behring, Germany.

### Assessment of reproductive parameters

Reproductive parameters were obtained by a personal interview by trained medical staff in the KORA S4 and F4 survey.

Age at menarche was defined as age at the first menstrual bleeding, assessed in full years. The question, which was asked in S4 was open-ended: “At what age did you have your first menstrual period (menarche)?” [20]. Women were asked in S4 and F4 to recall their age at their last menstruation, number of pregnancies, live-born children, if they ever used oral contraceptives or hormone replacement therapy. Women were defined postmenopausal at the absence of menses for 12 consecutive months. If the underlying reason for the absence of menses was a pregnancy or a lactation period, women were classified as premenopausal. Women were regarded as postmenopausal if they had bilateral oophorectomy (either alone or in combination with hysterectomy) and had hysterectomy without bilateral oophorectomy and were above 50 years in case there was no onset of menopause before hysterectomy. Women currently under hormone replacement therapy were stratified to the group of postmenopausal women in the analysis. Parity was defined as the number of deliveries, categorized in three groups: Nulliparae, 1–2 deliveries and three deliveries or more. On the basis of the classified medication data the categories “current use of oral contraceptives” and “current use of hormone replacement therapy” were created. Ever use of oral contraception or hormone replacement therapy was assessed in the interviews. Furthermore the women were asked about the presence of two symptoms of the menopausal transition: Hot flashes and depressive mood. Due to missing values and the difficulty to assign women an exact date of menopause, e.g. due to ongoing vaginal bleeding because of hormone replacement therapy or hysterectomy while still being premenopausal, this variable is only available for 693 women out of the 959 women classified as postmenopausal. The variable time since menopause was created by calculating the difference between age at menopause and current age.

### Statistical analyses

Basic characteristics of the study population were analyzed for the whole sample and stratified by quartiles of serum uric acid levels. The cut-offs are below 3.70 mg/dl for quartile 1 (Q1), 3.70 to 4.34 mg/dl (Q2), 4.34 to 5.15 mg/dl (Q3) and above 5.15 mg/dl (Q4). For normally distributed variables the mean and standard deviation and for non-normally distributed variables, the median and the interquartile range were given. For categorical variables percentages were calculated. For testing differences between uric acid categories, chi-square-tests for categorized outcome variables and regression models for linear outcomes were performed.

Means of uric acid levels are presented for grouped reproductive variables. Covariance-adjusted means were calculated. In the analyses three models were fitted, one controlling for age [years], the second model controlled for age [years] and BMI [kg/m^2^] and the third model controlled for the following variables: age [years], BMI [kg/m^2^], alcohol intake >20 g/day (yes/no), current use of uricosuric or uricostatic medication (yes/no) and serum creatinine [mg/dl]. The confounder were on one hand chosen due to literature research, which shows that age [10], BMI [11], [16] and alcohol consumption [16] is highly associated with serum uric acid levels. We included current use of uricosuric or uricostatic medication and serum creatinine, because of their direct influence on serum uric acid levels, furthermore we added current hypertension (yes/no), HDL cholesterol [mg/dl], total cholesterol [mg/dl], history of diabetes (yes/no) and education [years]. The analyses were performed for the whole sample and additionally stratified in the two groups of premenopausal and postmenopausal women. Included in the group of postmenopausal women were women currently under hormone therapy. General linear models were fitted using SAS procedure GLM.

Significance tests were two-tailed and p-values less than 0.05 were considered statistically significant. All analyses were performed using SAS (version 9.2, SAS Institute Inc, Cary, NC, USA).

## Results

Of the 1530 women included in the final analysis, 571 were classified premenopausal and 959 were classified postmenopausal. The mean age of all study participants was 55.7 years (standard deviation 13.1). The mean age of premenopausal women was 42.4 (5.8) years and of postmenopausal women 63.6 (9.2) years.

Characteristics of the study sample according to quartiles of serum uric acid levels are shown in Table 1. In summary, women with higher uric acid levels in comparison to the participants with lower uric acid levels were more likely to be older, have a higher BMI and waist circumference, a higher systolic and diastolic blood pressure, were more likely to suffer from hypertension, had higher mean serum creatinine levels, and lower HDL cholesterol levels. They were less physically active, were more likely to use uricosuric or uricostatic medication, were more likely to suffer from diabetes and reported less years of education. Women with higher uric acid levels had a lower age at menarche, reported more often a history of hysterectomy and more often the symptom of hot flashes during menopausal transition.

**Table 1:** Characteristics of the study population, mean (SD) and prevalences (%) of variables according to quartiles of uric acid, KORA F4 study (women 32–81 years).

	Uric Acid (mg/dl)					
	All probands	Q1:	Q2:	Q3:	Q4:	p-value
		<3.70	3.70 to <4.34	4.34 to <5.15	≥5.15	
n	1530	382	382	383	383	
Age (years)	55.7 (13.1)	50.3 (12.1)	53.2 (12.5)	57.0 (12.8)	62.2 (11.8)	<0.0001
BMI (kg/m^2^)	27.3 (5.3)	24.4 (4.0)	26.2 (4.6)	28.0 (4.9)	30.7 (5.5)	<0.0001
Waist circumference (cm)	88.1 (13.3)	80.3 (10.5)	85.1 (11.2)	90.0 (12.7)	97.1 (12.5)	<0.0001
Systolic blood pressure (mmHg)	117.2 (18.1)	112.5 (15.7)	115.0 (17.4)	117.9 (18.5)	123.2 (18.7)	<0.0001
Diastolic blood pressure (mmHg)	72.9 (9.4)	71.6 (9.2)	72.6 (8.6)	73.4 (9.3)	74.2 (10.3)	0.013
Actual hypertension (%)^a^	28.1	10.5	17.5	30.3	54.0	<0.0001
Serum creatinine (mg/dl)^b^	0.80 (0.71–0.89)	0.75 (0.66–0.82)	0.78 (0.70–0.85)	0.80 (0.72–0.89)	0.87 (0.77–0.95)	<0.0001
HDL cholesterol (mg/dl)	61.2 (14.3)	64.9 (14.0)	63.8 (13.9)	60.5 (14.4)	55.7 (12.9)	<0.0001
Total cholesterol (mg/dl)	218.4 (39.5)	208.9 (35.2)	217.2 (38.6)	220.3 (40.7)	227.1 (41.2)	<0.0001
Current use of uricosuric or uricostatic medication (%)	1.4	0.3	0.8	0.8	3.9	<0.0001
Current smoking (%)	15.4	16.8	17.2	17.0	10.4	0.542
Alcohol intake (>20 g/day in %)	14.8	16.0	16.2	13.1	13.8	0.816
Physically active (%)	55.6	61.4	56.9	53.5	50.7	0.154
History of diabetes (%)	5.9	2.4	2.3	5.2	13.8	<0.0001
Education ≥12 years (%)	27.8	36.2	31.6	25.6	17.8	<0.0001
Age at menarche (years)	13.5 (1.6)	13.6 (1.5)	13.5 (1.6)	13.4 (1.6)	13.5 (1.6)	0.338
Nulliparae (%)	14.8	16.5	15.1	16.4	11.0	0.989
Multiparae (≥3 deliveries, %)	36.4	32.0	36.3	36.0	41.3	0.104
Postmenopausal status (%)^c^	50.6	33.1	43.1	56.7	69.5	0.004
Age at menopause (years)^d^	48.9 (5.1)	48.5 (5.5)	49.0 (5.3)	48.6 (5.1)	49.2 (4.7)	0.297
Time since menopause (years)^d^	15.8 (9.2)	13.9 (8.9)	14.8 (9.2)	15.4 (9.5)	17.7 (8.8)	0.001
Current use of HRT (%)	9.0	6.3	11.0	8.4	10.2	0.698
Ever use of HRT (%)	41.7	35.4	39.7	44.1	47.5	0.454
Current use of OC (%)	6.5	10.2	6.8	7.0	1.8	0.706
Ever use of OC (%)	73.7	82.4	76.2	72.3	64.0	0.103
Hysterectomy (%)	22.3	15.2	18.8	21.9	33.2	0.036
Bilateral oophorectomy (%)	4.1	1.6	3.1	5.0	6.5	0.118
Hot flashes (%)	35.6	28.6	37.9	36.3	39.7	0.029
Depressive mood (%)	33.8	32.0	33.9	31.9	37.3	0.628

Abbreviations: Q: quartile, BMI: body mass index, SD: standard deviation, OC: oral contraceptives, HRT: hormone replacement therapy, HDL: high density lipoprotein.

Data are expressed as mean (SD) or percentages respectively.

p-value: chi-square test was performed to test the difference between the parameters and continuous uric acid levels.

a defined as use of antihypertensive medication, being aware of having hypertension or blood pressure values greater than 140/90 mmHg.

b due to the skewed distribution of creatinine, the median and interquartile range is shown.

c defined as postmenopausal women and women under hormone replacement therapy.

d n = 693, due to missing values of age at menopause.

Table 2 shows the association between reproductive parameters and mean uric acid levels for the total sample. After age-adjustment, menopausal status, lower age at menarche and the presence of hot flashes were significantly associated with higher mean uric acid levels. After full adjustment, including BMI, the association with hot flashes lost significance, but the association with age at menarche and menopause status remained significant. Ever use of oral contraceptives showed an association with uric acid levels after adjustment for BMI and multiple confounders, but not in the age-adjusted analysis.

**Table 2:** Mean uric acid levels stratified by reproductive parameters, the KORA F4 study (N = 1530).

	lsmean	CI		lsmean	CI		lsmean	CI		p-value*
**Age at menarche**	**<12 years**			**12–15 years**			**>15 years**			
	n = 119			n = 1250			n = 161			
Model 1	4.70	4,50	4.90	4.52	4.46	4.58	4.27	4.09	4.44	<0.001
Model 2	4.60	4.41	4.78	4.52	4.46	4.57	4.35	4.19	4.51	0.033
Model 3	4.58	4.41	4.75	4.52	4.46	4.57	4.36	4.21	4.51	0.036
**Parity**	**Nulliparae**			**1–2 deliveries**			**>2 deliveries**			
	n = 226			n = 747			n = 557			
Model 1	4.44	4.30	4.59	4.50	4.42	4.58	4.53	4.44	4.63	0.188
Model 2	4.51	4.38	4.65	4.51	4.44	4.58	4.49	4.41	4.58	0.946
Model 3	4.52	4.39	4.64	4.50	4.43	4.57	4.51	4.43	4.59	0.872
**Postmenopausal status**	**Yes**			**No**						
	n = 959			n = 571						
Model 1	4.61	4.52	4.70	4.33	4.20	4.46				0.003
Model 2	4.59	4.51	4.67	4.36	4.25	4.48				0.011
Model 3	4.60	4.52	4.68	4.34	4.23	4.46				0.002
**Current use of HRT**	**Yes**			**No**						
	n = 137			n = 1393						
Model 1	4.47	4.29	4.66	4.51	4.45	4.57				0.729
Model 2	4.55	4.37	4.72	4.50	4.45	4.55				0.626
Model 3	4.58	4.42	4.75	4.50	4.45	4.55				0.318
**Ever use of HRT**	**Yes**			**No**						
	n = 638			n = 892						
Model 1	4.45	4.36	4.54	4.54	4.47	4.62				0.122
Model 2	4.49	4.41	4.57	4.51	4.45	4.58				0.661
Model 3	4.51	4.43	4.59	4.50	4.43	4.56				0.815
**Current use of OC**	**Yes**			**No**						
	n = 99			n = 1431						
Model 1	4.41	4.19	4.64	4.51	4.45	4.57				0.419
Model 2	4.46	4.25	4.67	4.51	4.45	4.56				0.674
Model 3	4.40	4.20	4.60	4.51	4.46	4.56				0.293
**Ever use of OC**	**Yes**			**No**						
	n = 1128			n = 402						
Model 1	4.52	4.46	4.59	4.45	4.33	4.58				0.360
Model 2	4.54	4.48	4.61	4.40	4.28	4.51				0.033
Model 3	4.55	4.49	4.61	4.38	4.27	4.48				0.009
**Hysterectomy**	**Yes**			**No**						
	n = 341			n = 1189						
Model 1	4.61	4.49	4.73	4.47	4.41	4.54				0.060
Model 2	4.55	4.43	4.66	4.49	4.43	4.55				0.428
Model 3	4.55	4.45	4.66	4.49	4.44	4.55				0.154
**Bilateral oophorectomy**	**Yes**			**No**						
	n = 62			n = 1468						
Model 1	4.68	4.40	4.95	4.50	4.44	4.55				0.223
Model 2	4.60	4.35	4.86	4.50	4.45	4.55				0.443
Model 3	4.65	4.41	4.89	4.50	4.45	4.55				0.230
**Hot flashes**	**Yes**			**No**						
	n = 545			n = 985						
Model 1	4.58	4.49	4.68	4.46	4.39	4.53				0.038
Model 2	4.53	4.45	4.62	4.49	4.43	4.55				0.424
Model 3	4.56	4.48	4.64	4.48	4.42	4.54				0.126
**Depressive mood**	**Yes**			**No**						
	n = 517			n = 1013						
Model 1	4.50	4.40	4.60	4.51	4.44	4.58				0.904
Model 2	4.50	4.41	4.59	4.51	4.45	4.57				0.839
Model 3	4.51	4.43	4.59	4.50	4.44	4.56				0.907

Abbreviations: CI: Confidence Interval, BMI: Body mass index, lsmean: least square mean.

* the p-values (linear model) are reported for age at menarche and parity as continuous variable.

Model 1: adjusted for age (in years).

Model 2: adjusted for age (in years) and BMI (kg/m^2^).

Model 3: adjusted for age (in years), BMI (kg/m^2^), serum creatinine (mg/dl), current use of uricosuric or uricostatic medication (yes/no), alcohol consumption (>20 g/d in %), current hypertension (yes/no), HDL cholesterol (mg/dl), total cholesterol (mg/dl), history of diabetes (yes/no) and education (years).

Table 3 and Table 4 show the results for the stratified analyses. In premenopausal women (Table 3) earlier age at menarche was associated with higher uric acid levels after adjustment for age and additional adjustment for BMI. This association lost significance after multivariable adjustment. In postmenopausal women (Table 4), age at menarche and ever use of hormone replacement therapy were associated with higher uric acid levels after adjustment for age, but these associations did not remain significant after further adjustment. Ever use of oral contraceptives showed no association with uric acid levels after adjustment for age, but after further adjustment for BMI and multivariable adjustment, the association became significant.

**Table 3:** Mean uric acid levels stratified by reproductive parameters in premenopausal women, the KORA F4 study (total n = 571).

	lsmean	CI		lsmean	CI		lsmean	CI		p-value*
**Age at menarche**	**<12 years**			**12–15 years**			**>15 years**			
	n = 48			n = 488			n = 35			
Model 1	4.29	4.05	4.54	4.03	3.95	4.10	3.93	3.64	4.22	0.002
Model 2	4.15	3.93	4.38	4.03	3.96	4.10	4.01	3.74	4.27	0.048
Model 3	4.12	3.90	4.34	4.04	3.97	4.11	4.01	3.75	4.27	0.083
**Parity**	**Nulliparae**			**1–2 deliveries**			**>2 deliveries**			
	n = 110			n = 283			n = 178			
Model 1	4.04	3.88	4.21	4.04	3.94	4.14	4.04	3.91	4.17	0.814
Model 2	4.05	3.89	4.20	4.04	3.95	4.14	4.04	3.92	4.16	0.971
Model 3	4.05	3.90	4.20	4.04	3.94	4.13	4.05	3.93	4.16	0.977
**Current use of OC**	**Yes**			**No**						
	n = 99			n = 472						
Model 1	4.00	3.83	4.18	4.05	3.97	4.13				0.616
Model 2	4.03	3.87	4.19	4.05	3.97	4.12				0.834
Model 3	4.00	3.84	4.16	4.05	3.98	4.12				0.607
**Ever use of OC**	**Yes**			**No**						
	n = 533			n = 38						
Model 1	4.04	3.97	4.12	4.04	3.76	4.32				0.989
Model 2	4.05	3.98	4.11	3.99	3.74	4.25				0.688
Model 3	4.04	3.98	4.11	4.03	3.78	4.28				0.931

Abbreviations: CI: Confidence Interval, BMI: Body mass index, lsmean: least square means.

* the p-values (linear model) are reported for age at menarche and parity as continuous variable.

Model 1: adjusted for age (in years).

Model 2: adjusted for age (in years) and BMI (kg/m^2^).

Model 3: adjusted for age (in years), BMI (kg/m^2^), serum creatinine (mg/dl), current use of uricosuric or uricostatic medication (yes/no), alcohol consumption (>20 g/d in %), current hypertension (yes/no), HDL cholesterol (mg/dl), total cholesterol (mg/dl), history of diabetes (yes/no) and education (years).

**Table 4:** Reproductive parameters and uric acid levels in postmenopausal women, including women currently under hormone replacement therapy, the KORA F4 study (n = 959).

	lsmean	CI		lsmean	CI		lsmean	CI		p-value*
**Age at menarche**	**<12 years**			**12–15 years**			**>15 years**			
	n = 71			n = 762			n = 126			
Model 1	4.92	4.64	5.20	4.81	4.73	4.90	4.50	4.29	4.72	0.017
Model 2	4.86	4.60	5.12	4.80	4.72	4.88	4.59	4.39	4.79	0.178
Model 3	4.82	4.57	5.07	4.80	4.73	4.88	4.61	4.43	4.80	0.254
**Parity**	**Nulliparae**			**1–2 deliveries**			**>2 deliveries**			
	n = 116			n = 464			n = 379			
Model 1	4.64	4.42	4.86	4.77	4.66	4.88	4.83	4.71	4.96	0.142
Model 2	4.79	4.58	4.99	4.78	4.68	4.89	4.78	4.66	4.89	0.975
Model 3	4.80	4.61	5.00	4.77	4.68	4.87	4.78	4.68	4.89	0.801
**Current use of HRT**	**Yes**			**No**						
	n = 137			n = 822						
Model 1	4.69	4.48	4.90	4.80	4.71	4.88				0.355
Model 2	4.78	4.59	4.97	4.78	4.70	4.86				0.979
Model 3	4.81	4.63	4.99	4.78	4.70	4.85				0.730
**Ever use of HRT**	**Yes**			**No**						
	n = 551			n = 408						
Model 1	4.70	4.60	4.81	4.88	4.76	5.00				0.026
Model 2	4.74	4.65	4.84	4.83	4.72	4.94				0.238
Model 3	4.77	4.68	4.85	4.80	4.70	4.90				0.648
**Ever use of OC**	**Yes**			**No**						
	n = 595			n = 364						
Model 1	4.81	4.71	4.91	4.73	4.59	4.86				0.370
Model 2	4.85	4.75	4.94	4.67	4.54	4.79				0.034
Model 3	4.86	4.77	4.94	4.66	4.54	4.78				0.014
**Age at menopause** a	**<50 years**			**≥50 years**						
	n = 333			n = 360						
Model 1	4.83	4.70	4.96	4.85	4.72	4.98				0.814
Model 2	4.80	4.68	4.93	4.88	4.76	5.00				0.404
Model 3	4.82	4.70	4.94	4.86	4.75	4.98				0.593
**Time since menopause** a	**≤16 years**			**>16 years**						
	n = 370			n = 323						
Model 1	4.87	4.69	5.04	4.82	4.66	4.98				0.760
Model 2	4.87	4.72	5.02	4.81	4.65	4.97				0.645
Model 3	4.81	4.65	4.96	4.87	4.73	5.01				0.584
**Hysterectomy**	**Yes**			**No**						
	n = 341			n = 618						
Model 1	4.83	4.70	4.96	4.75	4.66	4.85				0.326
Model 2	4.78	4.66	4.90	4.78	4.69	4.87				0.979
Model 3	4.78	4.67	4.89	4.78	4.70	4.86				0.992
**Bilateral oophorectomy**	**Yes**			**No**						
	n = 62			n = 897						
Model 1	4.93	4.62	5.23	4.77	4.69	4.85				0.334
Model 2	4.85	4.57	5.14	4.78	4.70	4.85				0.600
Model 3	4.91	4.65	5.18	4.77	4.70	4.84				0.310
**Hot flashes**	**Yes**			**No**						
	n = 418			n = 541						
Model 1	4.84	4.72	4.96	4.74	4.63	4.84				0.229
Model 2	4.80	4.68	4.91	4.77	4.67	4.87				0.752
Model 3	4.80	4.70	4.91	4.76	4.67	4.85				0.569
**Depressive mood**	**Yes**			**No**						
	n = 351			n = 608						
Model 1	4.76	4.63	4.88	4.80	4.70	4.89				0.635
Model 2	4.75	4.63	4.87	4.80	4.71	4.89				0.514
Model 3	4.76	4.65	4.87	4.79	4.71	4.88				0.684

Abbreviations: CI: Confidence Interval, BMI: Body mass index, lsmean: least square means.

* the p-values (linear model) are reported for age at menarche and parity as continuous variable.

Model 1: adjusted for age (in years).

Model 2: adjusted for age (in years) and BMI (kg/m^2^).

Model 3: adjusted for age (in years), BMI (kg/m^2^), serum creatinine (mg/dl), current use of uricosuric or uricostatic medication (yes/no), alcohol consumption (>20 g/d in %), current hypertension (yes/no), HDL cholesterol (mg/dl), total cholesterol (mg/dl), history of diabetes (yes/no) and education (years).

a n = 693, due to missing values of age at menopause.

## Discussion

This study demonstrates that age at menarche and menopausal status are inversely associated with mean serum uric acid levels, even after adjustment for a variety of confounding variables. The association with age at menarche was stronger in premenopausal than in postmenopausal women, although significance was lost after multivariable-adjustment probably due to a lack of power in these stratified analyses. Ever use of oral contraceptives showed an association with uric acid levels after adjustment for BMI and multiple confounders. In stratified analyses, this relationship could be shown in postmenopausal women only.

Reproductive events which occur over the life-span of women are expected to influence hormonal levels, especially estrogen levels. Estrogens have an impact on the renal tubular handling of uric acid [18], [21], [22] and therefore are possibly explaining the underlying relation between some reproductive parameters and uric acid levels, suggesting that premenopausal levels of estrogen in women cause a greater renal clearance of uric acid [18], [21], [22], but the underlying mechanisms are not fully known [22]. Administration of estrogen to males decreases serum uric acid levels [22], which could be shown as well in transsexual persons under long-term treatment with sex steroids undergoing cross-sex hormone therapy [23]. Whether or not testosterone is of influence is not fully understood [23]. Therefore, one possible explanation for the gender difference could be the different estrogen levels in men and women, but this topic needs further research.

So far, the mechanisms underlying the association of age at menarche and uric acid levels are unclear. It is possible that early age at menarche only presents a marker for childhood obesity, which is known to induce earlier menarche. A recently published study shows that age at menarche is associated with diabetes, attenuated by adult and adolescent BMI, but less with BMI measured earlier in childhood [24]. Whether it acts in combination or as a risk factor by itself or through sex hormone changes over the life span needs further research. Existing data suggest that about half of the variance in the timing of puberty is due to genetic reasons [25], and there seems to be a genetic basis for the phenotypic associations between age at menarche and BMI [13]. Prospective studies are needed to understand the underlying mechanisms by which lower age at menarche increases uric acid levels in adulthood and whether there is a causal link to the development of chronic diseases.

Parity was not associated with uric acid levels in our study. Since parity was shown to be associated with cardiovascular disease [26] and the metabolic syndrome [27], [28], a different underlying causality could be assumed. Further studies confirming our results are needed.

Current use of oral contraceptives didn't show an association with uric acid levels, but ever use of oral contraceptives was independently associated with higher levels of uric acid. In stratified analyses this relationship was found in postmenopausal but not in premenopausal women.

Menopausal status was associated with uric acid levels after adjustment for age and multivariable-adjustment. This is in good agreement with other studies, which showed that the postmenopause is associated with higher uric acid levels [10], [11], independently of age. In a much larger study than the present one, Hak et al. examined 7662 women above age 20 and showed an independent association of menopause with higher serum uric acid levels. Postmenopausal hormone use was associated with lower uric acid levels [10]. The lack of association between hormone replacement therapy and uric acid levels in our study may be due to lack of power and might be due to the difference of the study sample: The mean age of premenopausal women in Hak's study was 34 years, which is much younger than the premenopausal women in our study. Another explanation might be a difference in the usage of the hormone replacement therapy. Hak's study was conducted between 1988 and 1994 in an US population [10], while our study sample was examined in 2006 to 2008 in South Germany, suggesting a different drug range, dosage and difference in administration. This possibly influences the results. Further research is needed to assess this in depth.

The lack of association between hysterectomy and uric acid levels in the present study is not surprising, since a hysterectomy does not cause a change in the hormone system. Contrary, bilateral oophorectomy leads to a surgical menopause and thus a change in the hormone system and therefore could possibly have an influence on uric acid levels, as it has been shown by Hak et al. [10]. In our study only 62 women had a bilateral oophorectomy, and some of them were already postmenopausal at the time of surgery, possibly explaining the lack of association.

Hot flashes and menopausal induced depressive mood were not associated with uric acid levels in our study. Only in the age-adjusted model of the whole population there seemed to be a slight association between hot flashes and uric acid levels, which needs to be assessed in further studies. After adjustment for BMI, this association was lost. Obesity has been described to be associated with fewer hot flashes [29]. One theory to explain this is that it is due to relatively elevated estrogen levels through the aromatization of estrogen in body fat. BMI is highly associated with uric acid levels [11], [16]. So if there is an effect, our data suggest that it is probably only a small one and other regulations are more dominant. The underlying physiology and risk factors for hot flashes have not been examined extensively and most is not known. So far, there is a lack of other studies investigating the association of hot flashes and symptoms during menopausal transition with uric acid levels.

### Strength and limitations

The cross-sectional design of the study represents a limitation, implicating that cause and effect relationships cannot be discerned. Furthermore, although we adjusted for a variety of important confounding variables, residual confounding cannot entirely be excluded. Since diet has an impact on uric acid levels it is a further limitation of our study that we could not adjust for dietary habits except for alcohol intake. Recall bias is another limitation. However, retrospective assessment of reproductive variables like age at menarche [30] and age at menopause [31] has been shown to be reliable and valid. Some reproductive factors in this study were defined fairly broad, like ever use of oral contraceptives or ever use of hormone replacement therapy, without adjustment for the timing or duration of the intake. Only asking for the presence of hot flashes and not taking into consideration the amount, severity and duration might as well lead to an underestimation of an association with uric acid levels.

The strengths of the study are the large number of females randomly drawn from the general population, and the availability of data on lifestyle and multiple risk factors, which were measured according to a standardized protocol.

Another limitation is the multiple testing situation. Since all our explaining variables are reproductive parameters which are somehow correlated with each other, simple correction of p-values would have been too conservative. We are aware, however, that in this situation our results can only be seen as explorative, showing a tendency, which have to be reproduced in bigger studies.

### Conclusion and Implication

Earlier age at menarche, postmenopausal status and a history of oral contraceptive use in postmenopausal women was associated with higher uric acid levels. Other reproductive factors, including parity, current or ever use of hormone replacement therapy, current use of oral contraceptives, hysterectomy, bilateral oophorectomy, hot flashes or depressive mood in relation to menopausal transition were not associated with uric acid levels.

Further studies, in particular prospective studies are needed to investigate the influence of reproductive parameters on serum levels of uric acid and finally on the manifestation of chronic diseases.
